# DNA Barcoding for Identification and Traceability in the Entire Industrial Chain of Lonicerae Japonicae Flos

**DOI:** 10.3390/plants15192968

**Published:** 2026-09-29

**Authors:** Ruoxing Liu, Zhijie Shi, Wenjie Xu, Yanfei Li, Jingyuan Song, Tianyi Xin

**Affiliations:** 1 Key Lab of Chinese Medicine Resources Conservation, State Administration of Traditional Chinese Medicine of the People’s Republic of China, Engineering Research Center of Chinese Medicine Resource, Ministry of Education, Institute of Medicinal Plant Development, Chinese Academy of Medical Sciences & Peking Union Medical College, Beijing 100193, China; 2 State Key Laboratory of Bioactive Substance and Function of Natural Medicines, Institute of Medicinal Plant Development, Chinese Academy of Medical Sciences & Peking Union Medical College, Beijing 100193, China

**Keywords:** Lonicerae Japonicae Flos, entire industrial chain, DNA barcoding, mini-barcode

## Abstract

As a traditional medicinal plant in China, Lonicerae Japonicae Flos (LJF) is extensively used in traditional Chinese medicine and Medicine Food Homology products, supporting a well-established industrial chain with diverse applications. However, the entire industrial chain is persistently compromised by adulteration with adulterants such as Lonicerae Flos (LF). Because LJF and LF are highly similar in appearance, misidentification can occur in trading and processing, and the substantial price gap of ¥94.0/kg for LJF, and ¥31.5/kg for LF, further increases incentives for substitution. Here, analytical approaches including morphological observation, DNA barcoding, and mini-barcode were integrated to identify 94 samples in the entire industrial chain, which included seeds, leaves, flower buds, decoction pieces, and traditional Chinese patent medicines (TCPMs). A total of 19 adulterants were detected among 72 commercial samples, including eight in 10 seed samples, two in 14 flower bud samples, four in 25 decoction pieces samples, and five in 23 TCPM samples. Morphological observation allowed efficient preliminary screening of upstream samples but was unsuitable for deeply processed products. Universal DNA barcoding could be used to identify diverse species, but DNA degradation compromised sequencing success in heavily processed materials. By comparison, mini-barcode achieved high identification efficiency for the heavily processed samples. These findings provided technical support for a quality assurance framework for the LJF industry. The framework could provide practical guidance for market regulation authorities and industry stakeholders to strengthen supplier auditing, batch release testing, and traceability from cultivation to the market.

## 1. Introduction

The global food and health landscape is undergoing a paradigm shift, with an increasing emphasis on the quality, safety, and efficacy of traditional and complementary medicines according to the World Health Organization [1], which are closely intertwined with Medicine Food Homology (MFH, materials for both food and medicine). Demand for herbal products positioned as natural and health-promoting has continued to increase [2,3]. The National Medical Products Administration of China had taken proactive steps by advocating for intensified safety supervision of Traditional Chinese Medicine (TCM), which is a vital component of the MFH. This included reinforced inspection measures for Chinese medicinal materials, decoction pieces, formula granules, and Traditional Chinese Patent Medicines (TCPMs), recognizing their significance in both therapeutic and potentially dietary supplement uses [4]. Within the domain of agricultural products, TCM represents a specialized and unique market segment. The industrial production process includes various stages such as planting, harvesting, storage, transportation, TCPM manufacturing, and marketing [5,6,7]. With the expansion of the demand for TCMs, the phenomenon of counterfeiting and adulteration in the market has become increasingly severe [8]. There is an urgent need to establish a comprehensive traceability system for quality control that encompasses the entire industrial chain from cultivation to market.

Lonicerae Japonicae Flos (LJF, honeysuckle) refers to the dried flower buds or early-bloomed flowers of *Lonicera japonica* Thunb. In previous studies, it was confirmed that LJF possessed significant medicinal value, including combating various viruses [9,10], regulating gut microbiota and inhibiting tumor growth [11,12], making it one of the most frequently utilized herbs for treating COVID-19 [13]. Meanwhile, it is recognized as a kind of dietary supplement according to the United States Pharmacopoeia. In Chinese daily life, it is widely consumed as herbal tea and a nutritive beverage, especially in summer to clear heat and remove toxins. 

In China, LJF is widely cultivated as an important medicinal and industrial crop. Its main production areas are distributed across several provinces. Among them, Pingyi County in Linyi City, Shandong Province, is one of the major production areas in the country. The planting area is approximately 433.34 km^2^, and the annual output of dried flowers is about 18 million kilograms [14]. In addition, Henan and Hebei are the two main production provinces of LJF in China, each having its own industrial characteristics: Henan Province is dominated by areas such as Xinxiang and Fengqiu, with a large planting scale and an active market [15]. Hebei Province has Julu County as its core production area, with stable product quality and a long history of cultivation, and holds an important position in the national supply of LJF. These production areas together form the core production belt of the Chinese LJF industry, supporting the stable development of downstream processing and the supply chain. Within the LJF industrial chain, the upstream encompasses cultivation bases and seedling propagation; the midstream focuses on processing and production. The primary processing methods for LJF include cleaning, plain stir-frying, and carbonization [16,17]. Plain stir-frying attenuates LJF’s cold property, thereby enhancing its suitability for individuals with deficiency syndromes; carbonization, in contrast, confers hemostatic properties [18]. Commercially available LJF decoction pieces are predominantly produced via cleaning and plain stir-frying—methods that preserve therapeutic integrity while ensuring safety and standardization. Stir-fried LJF is also widely consumed as a functional herbal tea, enjoying broad consumer acceptance. Downstream applications extend to TCPMs, cosmetics, functional foods, health-oriented beverages, and so on. The downstream consists of retail channels facing consumers, including traditional outlets such as pharmacies and hospitals as well as online platforms. Taking Pingyi County as an example, the local government has vigorously promoted intensive and deep processing of LJF, developing dozens of products, among which chain extension is most prominent in the herbal tea and pharmaceutical sectors. Accordingly, the Pingyi LJF industry has shifted from supplying only primary raw materials to an entire industrial chain development model.

LJF has a broad market, and it is included in more than 90 TCPM formulations [19]. However, LJF is more expensive than Lonicerae Flos (LF), which bears a resemblance to LJF and is derived from four species of *Lonicera*: *L. macranthoides*, *L. hypoglauca*, *L. confusa*, and *L. fulvotomentosa* [19]. With an average selling price of ¥94.0/kg for LJF versus ¥31.5/kg for LF [20], some unscrupulous vendors substitute LF for LJF for more profits, resulting in source-species confusion across multiple stages of the entire industrial chain [21]. It poses a potential threat to the safety and efficacy of clinical medications downstream of industrial production [22,23]. Moreover, downstream segments of the industrial chain lack a comprehensive quality control and traceability system, significantly hindering the standardization of production processes and applications.

**Figure 1:**
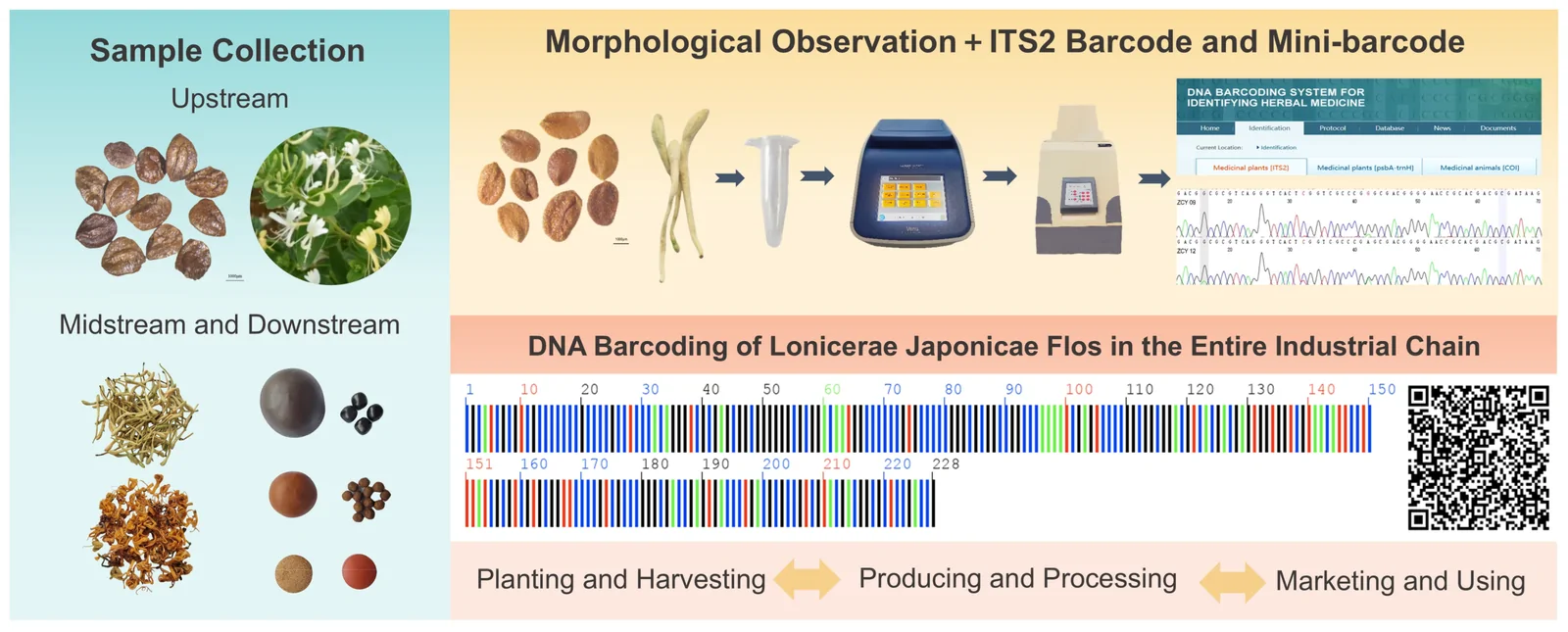
Research Framework for Species Identification and traceability of Lonicerae Japonicae Flos in the Entire Industrial Chain.

Currently, the existing identification techniques have not adequately covered the quality control needs of LJF in the entire industrial chain. It was difficult to distinguish the traits of closely related species of seeds, seedlings, and plants in the upstream. Identification often relies on a combination of multiple characters, which heavily depends on the integrity of the samples [24,25]. In the midstream and downstream, the morphological identification method of flower buds and medicinal materials relied heavily on examiner expertise and was therefore susceptible to misidentification, while extensive processing obscured or eliminated the morphological features of some decoction pieces and TCPMs. In addition, the method based on powder characteristics may prove unreliable due to morphological resemblance between LJF and other *Lonicera* species [26]. The physicochemical identification method was greatly affected by the external environment, soil, water, climate, and processing methods, consequently engendering inevitable limitations [27]. DNA barcoding targeted DNA sequences for detection and was characterized by rapid, accurate, and automated methods; Mini-barcode demonstrates high efficacy for samples with severe DNA degradation [28,29,30], with a reported minimum detectable LF adulteration ratio of approximately 6.2% [31]. Here, we aim to explore the effectiveness and stability of various methods, including morphological observation, DNA barcoding, and mini-barcode, from an entire industrial chain perspective, in the production process of LJF, with the goal of providing references for the quality control and traceability of LJF (Figure 1). 

## 2. Materials and Methods

### 2.1. Sample Collection

A total of 94 samples covering the entire industrial chain of LJF were collected using a convenience sampling strategy, including 11 seed samples and 21 leaf samples from the upstream, as well as 14 flower bud samples, 25 decoction piece samples, and 23 TCPM samples from the midstream and downstream. The samples were collected from 17 provinces in China, including Shandong, Henan, and Hebei. These three major production areas were recognized as the main geographical sources of LJF [32].

Among these, 22 authenticated voucher samples collected from different locations were used as reference standards, including one *L. japonica* seed sample (voucher No. ZZ01), 12 *L. japonica* leaf samples (voucher Nos. JYZW01–12), six *L. macranthoides* leaf samples (voucher Nos. JYZW13–18), two *L. hypoglauca* leaf samples (voucher Nos. JYZW19–20), and one *L. fulvotomentosa* leaf sample (voucher No. JWZW21). The voucher samples were authenticated by Prof. Yulin Lin from the Institute of Medicinal Plant Development (IMPLAD) and were deposited in IMPLAD. The leaf samples were dried with silica gel for preservation, whereas the seed samples were stored in a drying vessel for drying. 

In addition to the voucher samples, 72 commercially available samples were collected, all of which were readily available to consumers and patients. The seeds, flower buds and decoction pieces were purchased from different locations in dried form and covered the main production areas and markets of LJF. The TCPMs were purchased from major pharmacies in Beijing and from online platforms, with three to five batches collected for each TCPM. All LJF in the TCPMs were used as powder. Sample information, including sample types, locations and other details, is provided in Table 1. For molecular phylogenetic analysis, 21 ITS2 sequences of *L. japonica* and its adulterants were obtained from a previous study [33] (Table S1).

### 2.2. Morphological Observation

The commercial LJF seeds were observed under a stereomicroscope and compared with the standard sample of *L. japonica* seeds (ZZ01). The flower buds and decoction pieces were initially observed with the naked eye, and images were recorded using a camera. Samples that did not conform to the morphological description of LJF were further observed under a stereomicroscope. Morphological observation can characterize the appearance of a TCPM as a finished dosage form; however, in TCPMs subjected to grinding, extraction, and formulation, the botanical origin of an individual herbal ingredient generally cannot be reliably determined from appearance alone [31,34,35]. Therefore, morphological observation was not performed for these samples.

### 2.3. Genomic DNA Extraction and PCR Amplification and Sequencing 

For large seeds and those with hard shells, one seed was ground into powder with liquid nitrogen. The powder was then transferred to a 2.0 mL centrifuge tube. For small seeds, one seed was placed directly in a 2.0 mL centrifuge tube and crushed in a Mixer Mill MM400 (Retsch GmbH, Haan, Germany). For the leaves, flower buds, and decoction pieces that had been dried with silica gel, the outer surfaces were wiped with 75% ethanol. An amount of 20–30 mg of each was placed in a 2.0 mL centrifuge tube, which was then crushed in a Mixer Mill MM400 (Retsch GmbH, Haan, Germany). Genomic DNA was extracted using a Plant Genomic DNA Kit (Tiangen Biotech (Beijing) Co., Ltd., Beijing, China), and the samples were incubated in a 65 °C water bath for 30 min. 

For TCPMs, approximately 100 mg was directly placed in a 2.0 mL centrifuge tube, with 2–3 tubes prepared for each sample. 800 μL of pre-wash buffer (composition: 20 mmol·l^−1^ EDTA, pH 8.0; 100 mmol·l^−1^ Tris-HCl, pH 8.0; 700 mmol·l^−1^ NaCl; 2% PVP-40; Add 0.4% β-mercaptoethanol) was added, which was then vortexed for 5 min and centrifuged at 7500 rpm (Eppendorf, Hamburg, Germany) for 3 min. The supernatant was discarded, and these steps were repeated until the supernatant was almost colorless. Genomic DNA was extracted using a Plant Genomic DNA Kit (Tiangen Biotech (Beijing) Co., Ltd., Beijing, China), then incubated overnight in a 56 °C water bath.

Subsequent steps were carried out in accordance with the Chinese Pharmacopoeia [36]. The quality of the extracted genomic DNA was measured using a NanoDrop 2000 spectrophotometer (Thermo Fisher Scientific Inc., Waltham, MA, USA), and the samples were stored at −20 °C for future use.

The ITS2 region of all samples was amplified using the universal primers ITS2F (5′-ATGCGATACTTGGTGTGAAT-3′) and ITS3R (5′-GACGCTTCTCCAGACTACAAT-3′) [37]. The mini-barcode of TCPM samples and the heavily stir-fried LJF sample was targeted using the specific primers JYHF1 (5′-AGTGGTGGTCGTAACATTC-3′) and JYHR1 (5′-TCCTCCGCTTATTGATATGC-3′) [31]. For amplification, each 25 μL reaction contained approximately 20–100 ng of genomic DNA, 12.5 μL of 2 × Taq PCR MasterMix (Aidlab Biotechnologies Co., Ltd., Beijing, China), 1.0 μL of each primer (2.5 μM), and nuclease-free water to the final volume. All reactions were performed in a Veriti™ Thermal Cycler (Thermo Fisher Scientific Inc., Waltham, MA, USA). The reaction conditions for ITS2 region were as follows: 5 min at 95 °C followed by 40 cycles of 30 s at 94 °C, 30 s at 58 °C, and 45 s at 72 °C, with a final step of 10 min at 72 °C [33]. Reaction conditions for mini-barcode were the same as the ITS2 region, except for annealing at 56 °C for 30 s [31]. The PCR products were examined by electrophoresis on 1.5% agarose gels, and purified PCR products were sequenced on an ABI 3730XL sequencer (Thermo Fisher Scientific Inc., Waltham, MA, USA).

### 2.4. Data Analysis

Sanger sequencing chromatograms were proofread and assembled using the CodonCode Aligner V 12.0.4 (CodonCode Co., Centerville, MA, USA) software. The obtained ITS2 sequences were submitted to the DNA barcoding system for identifying herbal medicine (www.tcmbarcode.cn [accessed on 3 September 2026]) for BLAST alignment. Molecular phylogenetic analysis was conducted using MEGA 6, and a phylogenetic tree was constructed based on the K2P model, with a bootstrap repetition value of 1000. For mini-barcode, the species were identified using the SNP double peak method. Adulteration could be identified by analyzing the nucleotide composition at the two SNP sites. The expected bases were G and C for LJF, and A and T for LF, respectively [31].

## 3. Results

### 3.1. Morphological Features

**Figure 2:**
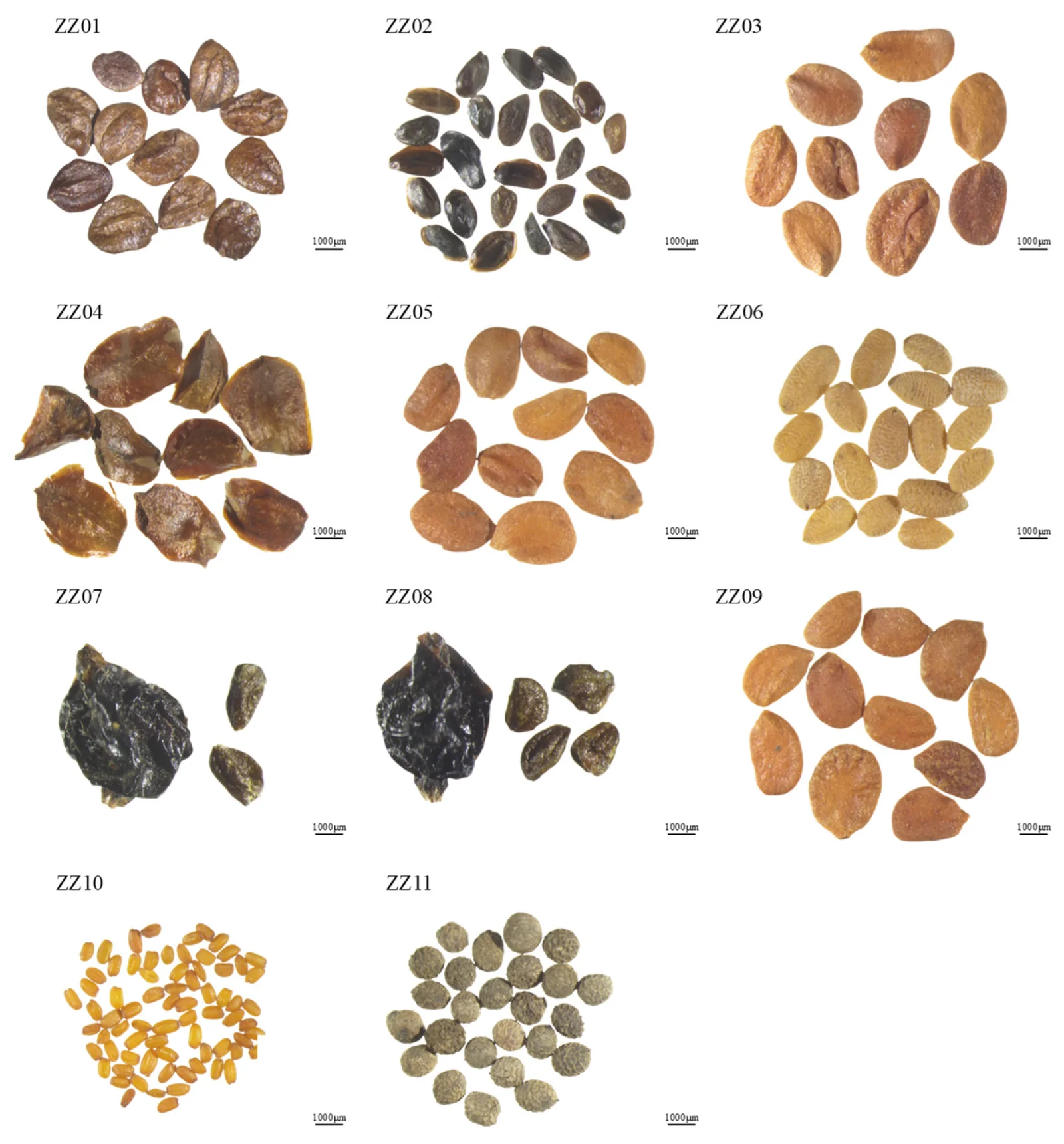
Morphological characteristics of collected seed samples. (ZZ01 was *Lonicera japonica*, ZZ07 and ZZ08 had the highest similarity to the morphological characteristics of ZZ01).

**Figure 3:**
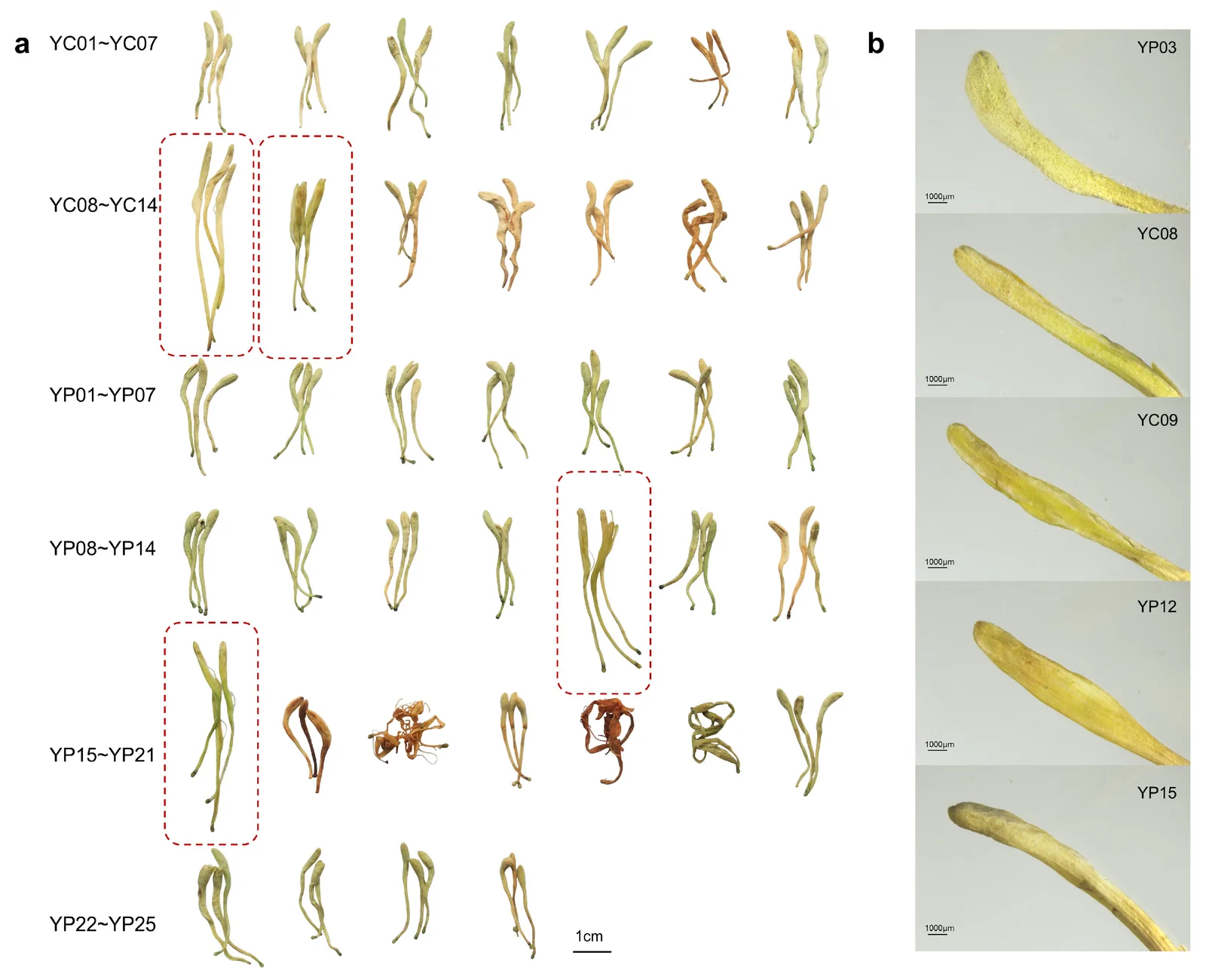
Morphological characteristics of collected flower buds and decoction pieces. (**a**) Observation of the appearance of commercial flower buds and decoction pieces. Four samples with obvious differences in appearance were marked. (**b**) Stereomicroscopic observation of the corolla heads of some samples. YP03 exhibited downy hairs, whereas the other samples displayed smooth surfaces devoid of such hairs.

The shape, size, color, and other characteristics of the seeds were observed and recorded by stereomicroscopy, detailed in Table S2. Among them, ZZ07 and ZZ08 were fruit-containing seeds after peeling, with the highest similarity to the morphological characteristics of LJF seeds (Figure 2). In the identification process for flower buds and decoction pieces, YP17, YP19, and YP20 showed evident signs of deep processing that complicated their identification. Notably, YC08, YP12, and YP15 displayed significantly greater lengths compared to other samples; moreover, YC08, YC09, YP12, and YP15 possessed smooth surfaces devoid of downy hairs (Figure 3a), a feature that was even more pronounced under stereomicroscopy (Figure 3b). Consequently, whether YC08, YC09, YP12, and YP15 were LJF required further verification using DNA barcoding analysis.

### 3.2. Species Identification Based on ITS2 Barcode 

**Figure 4:**
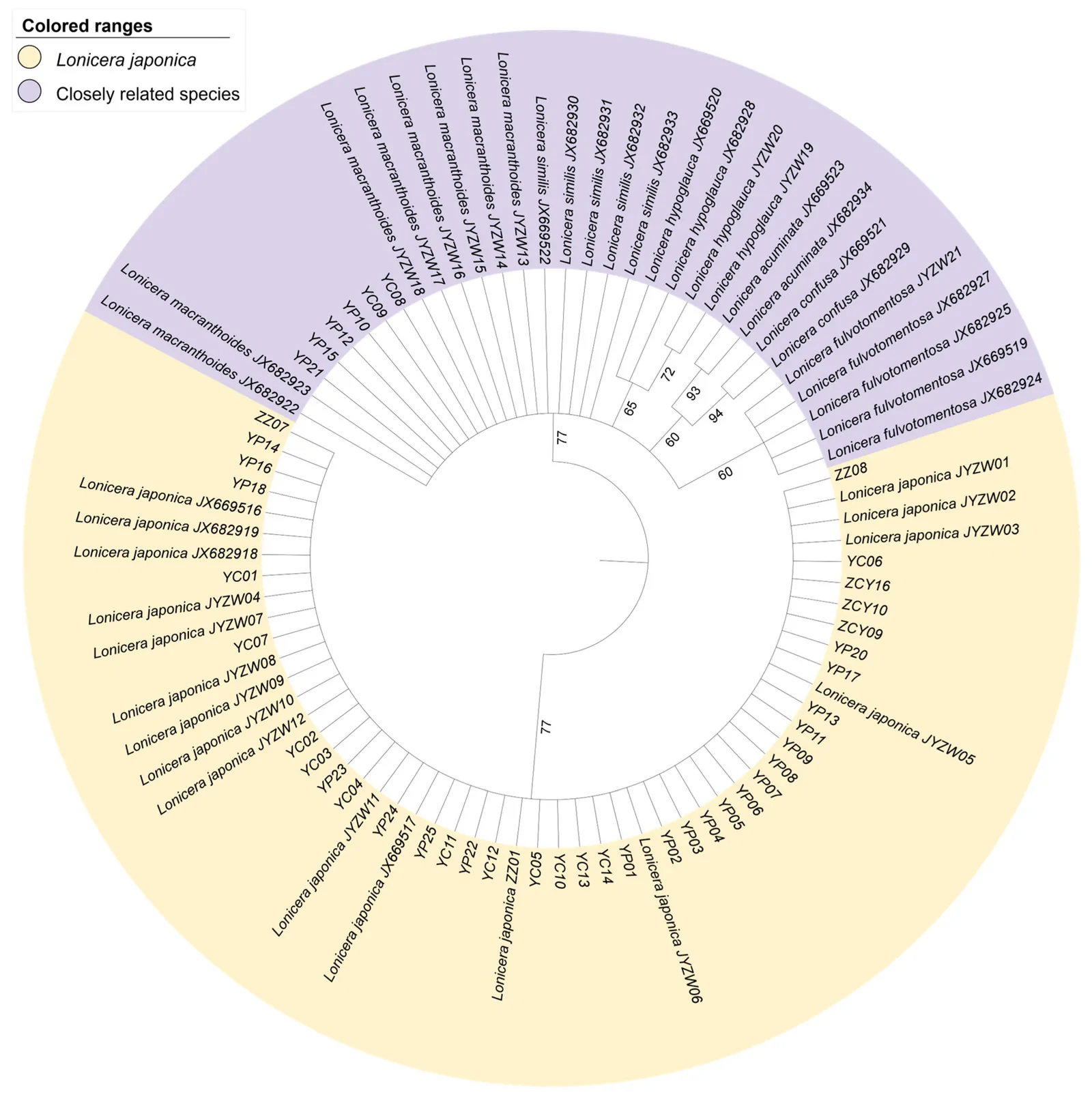
Phylogenetic tree of the entire industrial chain of *Lonicera japonica* and its adulterants constructed with ITS2 sequences using the NJ method. The bootstrap scores (1000 replicates) are shown (≥50%) for each branch.

Genomic DNA was successfully extracted from 94 samples, with DNA concentrations detailed in Table S3. Among the 94 samples, ITS2 sequences were successfully amplified in 84 samples, with amplicon lengths of approximately 500 bp (Figure S1). Sequencing was successful for 76 samples, and the ITS2 sequence length of LJF was 228 bp. The decoction pieces fried LJF (YP19) showed amplification failure. The TCPM samples exhibited varying rates of amplification and sequencing failures (Table 1). Among the 23 TCPM samples, the ITS2 sequences were successfully amplified in 14 samples, while six samples were successfully sequenced and readable sequences were obtained. The 76 successfully sequenced samples were subsequently submitted to the DNA barcoding system for identifying herbal medicine (www.tcmbarcode.cn) for BLAST alignment. Among them, 65 sequences were identified as LJF or LF (Table 1), and BLAST alignment percentages and scores are provided in Table S3. It’s demonstrated that LJF can be clearly distinguished from common adulterants (Figure 4). For commercial sample analysis, a total of 14 counterfeit samples were detected through the ITS2 barcode, with samples belonging to the *Lonicera* genus and other families. These counterfeit samples included eight of 10 commercial seed samples, two of 14 flower bud samples, and four of 24 decoction piece samples that could be successfully sequenced (Table 1).

### 3.3. Species Identification Based on Mini-Barcode

The mini-barcode fragments of LJF were effectively amplified from all 24 samples, including 23 TCPM samples and the one stir-fried LJF (YP19) that failed in ITS2 region amplification (Figure S2). All 24 samples were successfully amplified and sequenced. The length of the mini-barcode was approximately 110 bp. By analyzing the two SNP sites, YP19 was identified as *L. japonica*, and five out of the 23 TCPM samples showed varying degrees of adulteration (Figure S3). 

## 4. Discussion

### 4.1. DNA Barcoding Is an Important Tool for Quality Monitoring in the Entire Industrial Chain of LJF

Previous studies have provided a solid foundation for the molecular identification of LJF. For instance, Sun et al. systematically evaluated seven candidate DNA barcoding regions to identify LJF leaves and flower buds and found that the *psbA*-*trnH* region effectively discriminated LJF from closely related species [38]. Hou et al. subsequently examined flower buds and confirmed the stability and accuracy of ITS2 and *psbA*-*trnH* regions for distinguishing LJF from related species. They also reported that the 56 °C annealing temperature used by Sun et al. was unsuitable for amplifying the ITS2 region, given LJF’s high average GC content [33]. To address the severe DNA degradation and complex composition associated with TCPMs, Gao et al. developed a mini-barcode for LJF and subsequently extended its application to TCPMs [31]. Nevertheless, they focused on leaves, flower buds, decoction pieces, or TCPMs rather than the entire industrial chain. In this study, the application of DNA barcoding in the quality control of the entire industrial chain of LJF was reported for the first time. A total of 94 samples were analyzed, comprising seeds and leaves from the upstream, and different kinds of samples from the midstream and downstream, including flower buds, decoction pieces, and TCPMs. For the first time, seeds of LJF were incorporated into the identification research using DNA barcoding. Identification was based primarily on the ITS2 region, with morphological observation used for preliminary screening when morphological traits were retained. Mini-barcode was applied to highly processed samples with severely degraded DNA. This strategy contributed to establishing an integrated DNA barcoding workflow spanning the upstream, midstream, and downstream of the entire industrial chain for LJF, thereby substantially expanding the application of DNA barcoding to LJF identification and traceability and providing a technical basis for quality control throughout the entire industrial chain.

### 4.2. Critical Points for Applying DNA Barcoding in the Entire Industrial Chain of LJF

The acquisition of high-quality DNA from diverse sample types represents a critical initial step. DNA integrity, purity, and amplifiability directly affect PCR amplification and sequencing outcomes and also inform the selection of an appropriate barcode. Samples with high-quality DNA were suitable for ITS2 barcode analysis, whereas the shorter amplicon of the mini-barcode made it more suitable for samples with extensively degraded DNA. The length of the ITS2 region of LJF was 228 bp. In this study, it was successfully amplified from seeds, leaves, and flower buds but failed in one decoction piece sample, YP19, which had been fried. Among the 23 TCPM samples, the ITS2 region was successfully amplified in 14 samples. Among these, six samples were sequenced successfully and readable sequences were obtained. By contrast, the mini-barcode was successfully obtained from sample YP19 and all 23 TCPM samples, with a length of approximately 110 bp. In terms of DNA concentration, there were certain trends among the samples. In particular, the single-seed sampling strategy led to low DNA yield due to the limited starting material, while the leaf samples JYZW13, JYZW19, and JYZW20 exhibited low DNA concentrations, likely attributable to their extended storage time. However, no consistent decline in DNA concentration was observed with increasing processing intensity in the entire industrial chain. The overall differences may arise from the combined effects of several factors, such as sample type, storage duration, input quantity, and extraction pretreatment. In addition, concentration estimates obtained from absorbance measurements may be affected by coextracted compounds and do not necessarily reflect DNA integrity or suitability for amplification. For example, the TCPM samples ZCY02 and ZCY05 exhibited relatively high DNA concentrations but low purity and failed to yield ITS2 amplicons. DNA quality should therefore be evaluated comprehensively according to concentration, purity, integrity, and amplification performance rather than concentration alone. The application of thorough grinding, pre-wash buffer pretreatment, and overnight incubation at 56 °C represents a common and established improvement strategy for DNA extraction [36,39,40,41].

In terms of the species range of this study, the ITS2 region contained more informative sites and was therefore preferable for identifying unknown species in commercial samples, whereas mini-barcode was better suited for detecting mixed species originating from complex composition samples. Using the ITS2 region, not only *L. japonica*, the original species of LJF, was found in upstream samples, but also distantly related species, such as *Platycodon grandiflorus*, *Sambucus williamsii*, and *Descurainia sophia,* were detected in the commercial samples labeled as LJF seeds. Furthermore, the identification of commercially available samples under various conditions can also be complemented by the integration of other approaches. For commercial sample batches containing mixed adulterants, the point sampling method should precede analysis, including Sanger sequencing [42]. For complex mixtures that cannot be readily separated, pooled templates may generate superimposed Sanger sequencing chromatograms. In such cases, second-generation metabarcoding or third-generation single-molecule real-time (SMRT) sequencing could facilitate the simultaneous detection of multiple biological components [40,43].

DNA barcoding revealed adulteration at multiple stages in the entire industrial chain of LJF. In the upstream, samples generally retained relatively intact morphological structures. However, morphological characteristics alone were insufficient for exact species identification and therefore required confirmation using DNA barcoding. Eight of 10 commercial seed samples were inconsistent with the label name of LJF, and several belonged to species outside the genus *Lonicera*. In the midstream, certain morphological characteristics of flower buds and decoction pieces could support preliminary screening. However, similarities in morphology among related species and the loss of morphological features during processing limited accurate identification. Using DNA barcoding, we identified LF in two of 14 flower bud samples and four of 25 decoction piece samples. In the downstream, TCPM samples lacked complete morphological characteristics, and their DNA was frequently degraded. Therefore, mini-barcode analysis was particularly important. Of the 23 TCPM samples, five were found to be adulterated with LF. The Chinese Pharmacopoeia recognizes LJF and LF as distinct traditional Chinese medicinal materials derived from different botanical sources [19]. Accordingly, LF cannot be used as a substitute in products labeled as LJF. Based on the above information, DNA barcoding has substantial practical value for detecting species substitution and adulteration in the entire industrial chain of LJF.

### 4.3. Applications and Future Prospects of LJF Identification in the Entire Industrial Chain

Based on these findings, we established a tiered identification workflow covering the entire LJF industrial chain. This workflow could provide a practical technical framework for Chinese herbal medicine manufacturing enterprises, pharmaceutical inspection agencies, drug supervisory administrations, and hospitals to identify LJF. In the upstream cultivation stage, morphological observation should be combined with DNA barcoding to confirm the species identity of seeds, seedlings, and cultivated plants before planting. In the midstream production and downstream market stages, morphology could serve as a rapid prescreening tool, with DNA barcoding as a confirmatory step for samples suspected of adulteration. For deeply processed products in midstream and downstream, mini-barcode should be the preferred identification approach. Relevant institutions could combine identification results with supplier information, batch identifiers, and processing records to maintain continuous documentation of species identity during raw material input, production, finished product inspection, and supply chain traceability. This integration would improve the reliability and consistency of quality control in the entire industrial chain of LJF.

Building on our team’s long-term research on DNA barcoding, we have further developed a method to expand the scope of species identification targets through Analysis of whole-GEnome (AGE) [44,45,46]. The ITS2 barcode and mini-barcode are themselves components of the whole-genome, whereas AGE enables the systematic screening of potential targets throughout the genome, thereby providing a richer set of candidate targets for the precise discrimination of closely related species. Our team has published the chromosome-level whole-genome of *L. japonica* [47], while Yin et al. assembled the whole-genome of *L. macranthoides* [48]. These genomic resources provided a foundation for systematically characterizing genetic variation among species of *Lonicera*. Artificial intelligence could also facilitate the analysis of large comparative genomic datasets and accelerate the identification of genomic targets specific to individual species [49]. Future studies could adopt the evaluation framework established in this study to assess the applicability of relevant targets and their associated detection methods in different sample types in the entire industrial chain of LJF, including seeds, flower buds, decoction pieces, and TCPMs. Guided by the industrial demand for rapid, accurate, and economical authentication, new perspectives could be provided for LJF identification in the entire industrial chain.

## 5. Conclusions

In conclusion, by applying integrated methods, we collected 94 samples in the entire industrial chain of LJF and identified 19 adulterates in 72 commercially available samples, comprising eight of 10 commercial seed samples, two of 14 flower bud samples, four of 25 decoction pieces samples, and five of 23 TCPM samples. It was demonstrated that morphological observation could serve as an efficient preliminary screening tool for upstream samples but remained ineffective for deeply processed products. Furthermore, while the universal ITS2 barcode could detect a broad range of species, it suffered from low sequencing success in deeply processed products due to DNA degradation. Conversely, mini-barcode proved highly effective for these deeply processed products. Ultimately, this integrated approach facilitates the establishment of a comprehensive traceability system, which can support the development of a scientific regulatory framework and ensure the quality of products in practical production.

## Figures and Tables

**Table 1:** Samples of Lonicerae Japonicae Flos in the Entire Industrial Chain.

Chain Link	Sample Types	Sample No.	Collected Samples	Location	Nearest Match (www.tcmbarcode.cn/china [accessed on 3 September 2026])
Upstream	Seed	ZZ01	*Lonicera japonica*	Linyi, Shandong	*L. japonica*
		ZZ02	Seeds of LJF	Suqian, Jiangsu	*Platycodon grandiflorus*
		ZZ03	Seeds of LJF	Nanjing, Jiangsu	*Lonicera subsessilis*/*L. maackii*
		ZZ04	Seeds of LJF	Suqian, Jiangsu	*Juniperus virginiana* var. *silicicola*/*J. scopulorum*/*J. maritima*
		ZZ05	Seeds of LJF	Jiuquan, Gansu	*L. maackii*
		ZZ06	Seeds of LJF	Baoding, Hebei	*Sambucus williamsii*
		ZZ07	Seeds of LJF	Baoding, Hebei	*L. japonica*
		ZZ08	Seeds of LJF	Baoding, Hebei	*L. japonica*
		ZZ09	Seeds of LJF	Tongren, Guizhou	*Lonicera subsessilis*/*L. maackii*
		ZZ10	Seeds of LJF	Heze, Shandong	*Descurainia sophia*
		ZZ11	Seeds of LJF	Linyi, Shandong	*Eschscholzia californica* subsp. *mexicana*/*E. californica* subsp. *californica*
	Leaf	JYZW01-06	*L. japonica*	Nanjing, Jiangsu	*L. japonica*
		JYZW07-12	*L. japonica*	Beijing	*L. japonica*
		JYZW13	*L. macranthoides*	Shandong, Linyi	*L. macranthoides*/*L. hypoglauca*/*L. fulvotomentosa*
		JYZW14-18	*L. macranthoides*	Nanjing, Jiangsu	*L. macranthoides*/*L. hypoglauca*/*L. fulvotomentosa*
		JYZW19-20	*L. hypoglauca*	Nanning, Guangxi	*L. hypoglauca*
		JYZW21	*L. fulvotomentosa*	Baise, Guangxi	*L. fulvotomentosa*
Midstream and downstream	Flower bud	YC01	Flower buds of LJF	Linyi, Shandong	*L. japonica*
		YC02	Flower buds of LJF	Linyi, Shandong	*L. japonica*
		YC03	Flower buds of LJF	Fengqiu, Henan	*L. japonica*
		YC04	Flower buds of LJF	Linyi, Shandong	*L. japonica*
		YC05	Flower buds of LJF	Luan, Anhui	*L. japonica*
		YC06	Flower buds of LJF	Shiyan, Hubei	*L. japonica*
		YC07	Flower buds of LJF	Anqing, Anhui	*L. japonica*
		YC08	Flower buds of LJF	Bazhong, Sichuan	*L. macranthoides*/*L. hypoglauca*/*L. fulvotomentosa*
		YC09	Flower buds of LJF	Huaihua, Hunan	*L. macranthoides*/*L. hypoglauca*/*L. fulvotomentosa*
		YC10	Flower buds of LJF	Linyi, Shandong	*L. japonica*
		YC11	Flower buds of LJF	Linyi, Shandong	*L. japonica*
		YC12	Flower buds of LJF	Linyi, Shandong	*L. japonica*
		YC13	Flower buds of LJF	Linyi, Shandong	*L. japonica*
		YC14	Flower buds of LJF	Linyi, Shandong	*L. japonica*
	Decoction pieces	YP01	Decoction pieces of LJF	Linyi, Shandong	*L. japonica*
		YP02	Decoction pieces of LJF	Fengqiu, Henan	*L. japonica*
		YP03	Decoction pieces of LJF	Linyi, Shandong	*L. japonica*
		YP04	Decoction pieces of LJF	Fengqiu, Henan	*L. japonica*
		YP05	Decoction pieces of LJF	Linyi, Shandong	*L. japonica*
		YP06	Decoction pieces of LJF	Bozhou, Anhui	*L. japonica*
		YP07	Decoction pieces of LJF	Linyi, Shandong	*L. japonica*
		YP08	Decoction pieces of LJF	Linyi, Shandong	*L. japonica*
		YP09	Decoction pieces of LJF	Xinmi, Henan	*L. japonica*
		YP10	Decoction pieces of LJF	Linyi, Shandong	*L. macranthoides*/*L. hypoglauca*/*L. fulvotomentosa*
		YP11	Decoction pieces of LJF	Shangqiu, Henan	*L. japonica*
		YP12	Decoction pieces of LJF	Tongren, Guizhou	*L. macranthoides*/*L. hypoglauca*/*L. fulvotomentosa*
		YP13	Decoction pieces of LJF	Xinxiang, Henan	*L. japonica*
		YP14	Decoction pieces of LJF	Linyi, Shandong	*L. japonica*
		YP15	Decoction pieces of LJF	Shaoyang, Hunan	*L. macranthoides*/*L. hypoglauca*/*L. fulvotomentosa*
		YP16	Stir-fried LJF	Linyi, Shandong	*L. japonica*
		YP17	Stir-fried LJF	Xingtai, Hebei	*L. japonica*
		YP18	Stir-fried LJF	Linyi, Shandong	*L. japonica*
		YP19	Stir-fried LJF	Linyi, Shandong	*L. japonica*
		YP20	Stir-fried LJF	Xingtai, Hebei	*L. japonica*
		YP21	Decoction pieces of LJF	Fengqiu, Henan	*L. macranthoides*/*L. hypoglauca*/*L. fulvotomentosa*
		YP22	Decoction pieces of LJF	Guilin, Guangxi	*L. japonica*
		YP23	Decoction pieces of LJF	Xinxiang, Henan	*L. japonica*
		YP24	Decoction pieces of LJF	Julu, Hebei	*L. japonica*
		YP25	Decoction pieces of LJF	Shandong	*L. japonica*
	Traditional Chinese patent medicine	ZCY01	Zhizi Jinhua Pills	Chifeng, Inner Mongolia	-
		ZCY02	Zhizi Jinhua Pills	Chifeng, Inner Mongolia	-
		ZCY03	Zhizi Jinhua Pills	Beijing	-
		ZCY04	Zhizi Jinhua Pills	Beijing	Mixture of multiple species
		ZCY05	Zhizi Jinhua Pills	Jining, Shandong	-
		ZCY06	Yinqiao Jiedu Tablets	Beijing	Mixture of multiple species
		ZCY07	Yinqiao Jiedu Tablets	Beijing	Mixture of multiple species
		ZCY08	Yinqiao Jiedu Tablets	Kunming, Yunnan	-
		ZCY09	Yinqiao Jiedu Tablets	Foshan, Guangdong	*L. japonica*
		ZCY10	Jiannao Bushen Pills	Zibo, Shandong	*L. japonica*
		ZCY11	Jiannao Bushen Pills	Zibo, Shandong	-
		ZCY12	Jiannao Bushen Pills	Liaocheng, Shandong	-
		ZCY13	Niuhuang Qinggong Pills	Beijing	*Nelumbo nucifera*
		ZCY14	Niuhuang Qinggong Pills	Beijing	Mixture of multiple species
		ZCY15	Niuhuang Qinggong Pills	Jilin, Jilin	-
		ZCY16	Jinsang Sanjie Pills	Xi’an, Shaanxi	*L. japonica*
		ZCY17	Jinsang Sanjie Pills	Xi’an, Shaanxi	Mixture of multiple species
		ZCY18	Jinsang Sanjie Pills	Xi’an, Shaanxi	-
		ZCY19	Qingguo Pills	Beijing	Mixture of multiple species
		ZCY20	Qingguo Pills	Baoji, Shaanxi	*Paeonia lactiflora*
		ZCY21	Qingguo Pills	Shijiazhuang, Hebei	-
		ZCY22	Qingguo Pills	Hohhot, Inner Mongolia	-
		ZCY23	Qingguo Pills	Weinan, Shaanxi	*P. lactiflora*

“-” indicates not applicable.

## Data Availability

The original contributions presented in this study are included in the article/Supplementary Material. Further inquiries can be directed to the corresponding author.

## Supplementary Materials

The following supporting information can be downloaded at: https://www.mdpi.com/article/10.3390/plants15192968/s1, Figure S1: Agarose gel electrophoresis showing PCR amplification products of the ITS2 sequence; Figure S2: Agarose gel electrophoresis showing PCR amplification products of mini-barcode; Figure S3: Identification using mini-barcode combined with SNP double peak method; Table S1: ITS2 sequences used for molecular phylogenetic analysis; Table S2: Morphological characteristics of the collected seeds; Table S3: DNA Concentrations and BLAST results of samples in the Entire Industrial Chain.
