# Supplementary material for: DNA Barcoding for Identification and Traceability in the Entire Industrial Chain of Lonicerae Japonicae Flos
Source: Plants (Basel). 2026 Sep 29;15(19):2968. doi: 10.3390/plants15192968 (PMC13666573; doi:10.3390/plants15192968)
Supplement: Supplementary file 1 [file plants-15-02968-s001.zip › plants-4515679-supplementary.pdf]

Table S1. ITS2 sequences used for molecular phylogenetic analysis

Objects	GenBank No.
Lonicera japonica	JX682918
	JX682919
	JX669516
	JX669517
L. macranthoides	JX682922
	JX682923
L. confusa	JX682929
	JX669521
L. fulvotomentosa	JX682924
	JX682925
	JX689519
	JX682927
L. hypoglauca	JX682928
	JX669520
L. similis	JX682930
	JX682931
	JX682932
	JX682933
	JX669522
L. acuminata	JX682934
	JX669523

Table S2. Morphological characteristics of the collected seeds

Sample No.	Shape	Size	Color	Other characteristics
ZZ01	obovate or oval	1.9~2.6 mm long, 1.4~2.0 mm wide.	Blackish-brown or brow	Inverted "U"-shaped longitudinal groove on each dorsal and ventral surface
ZZ02	oval	1.5~2.3 mm long, 0.7~1.3 mm wide.	Black or brownish-black	Smooth, densely covered with black stripes, and glossy
ZZ03	Oblate oval	2.5~3.6 mm long, 1.9~2.4 mm wide.	Reddish-brown or yellowish-brown	With honeycomb-like tiny shallow pits
ZZ04	Irregular round shape	2.9~3.9 mm long, 1.5~2.8 mm wide.	Brownish.	With a hard outer shell; uneven, with a raised middle part
ZZ05	Oblate oval	2.5~3.0 mm long, 1.5~2.8 mm wide.	Reddish-brown or yellowish-brown.	With honeycomb-like tiny shallow pits
ZZ06	oblong	1.6~3.0mm long, 1.1~1.5 mm wide.	Yellow or light yellow	With a reticulated surface
ZZ07	Obovate	2.5~2.9 mm long, 1.2~1.5 mm wide.	Brownish-brown	Inverted "U"-shaped longitudinal groove on each dorsal and ventral surface
ZZ08	Obovate	1.9~2.4 mm long, 1.3~1.7 mm wide.	Brownish-brown	Inverted "U"-shaped longitudinal groove on each dorsal and ventral surface
ZZ09	Oblate ova	2.5~3.4 mm long, 1.5~2.5 mm wide.	Reddish-brown or yellowish-brown	With honeycomb-like tiny shallow pits

ZZ10	oblong	0.8~1.1 mm long, 0.3~0.5 mm wide.	Yellowish-brown or yellow	Slightly glossy surface with two longitudinal grooves, one more distinct.
ZZ11	Nearly spherical	Approximately 1.2 mm diameter	Black or gray	With a reticulated surface

Table S3. DNA Concentrations and BLAST results of samples in the Entire Industrial Chain

Sample No.	Collected samples	Lot No.	DNA concentration (ng/μL)	A260/A280	Nearest match (www.tcmbarcode.cn/china [accessed on 3 September 2026])	Similarity (%)	Score
ZZ01	*Lonicera japonica*	-	10.5	1.44	*Lonicera japonica*	100.0	442
ZZ02	Seeds of LJF	-	15.6	1.56	*Platycodon grandiflorus*	100.0	517
ZZ03	Seeds of LJF	-	21.6	1.80	*Lonicera subsessilis*	100.0	448
					Lonicera maackii	100.0	448
ZZ04	Seeds of LJF	-	70.4	1.99	*Juniperus virginiana* var. *silicicola*	94.5	347
					J. scopulorum	94.5	347
					J. maritima	94.5	347
ZZ05	Seeds of LJF	-	19.6	1.77	*L. maackii*	100.0	452
ZZ06	Seeds of LJF	-	45.2	2.03	*Sambucus williamsii*	100.0	448
ZZ07	Seeds of LJF	-	116.3	1.84	*L. japonica*	100.0	452
ZZ08	Seeds of LJF	-	145.8	1.97	*L. japonica*	100.0	452
ZZ09	Seeds of LJF	-	33.4	1.77	*Lonicera subsessilis*	100.0	448
					L. maackii	100.0	448
ZZ10	Seeds of LJF	-	11.0	1.97	*Descurainia sophia*	100.0	385
ZZ11	Seeds of LJF	-	75.9	1.99	*Eschscholzia californica* subsp. *mexicana*	100.0	472
					E. californica subsp. *californica*	100.0	472
JYZW01	*L. japonica*	-	1057.4	2.11	*L. japonica*	100.0	452
JYZW02	*L. japonica*	-	1987.8	2.13	*L. japonica*	100.0	452
JYZW03	*L. japonica*	-	374.7	2.09	*L. japonica*	100.0	452
JYZW04	*L. japonica*	-	913.0	2.12	*L. japonica*	100.0	452
JYZW05	*L. japonica*	-	840.0	2.14	*L. japonica*	100.0	452
JYZW06	*L. japonica*	-	1167.4	2.12	*L. japonica*	100.0	452
JYZW07	*L. japonica*	-	1540.8	2.11	*L. japonica*	100.0	452
JYZW08	*L. japonica*	-	1898.8	2.12	*L. japonica*	100.0	452
JYZW09	*L. japonica*	-	1401.0	2.11	*L. japonica*	100.0	452

JYZW10	*L. japonica*	-	1099.1	2.09	*L. japonica*	100.0	452
JYZW11	*L. japonica*	-	1798.6	2.10	*L. japonica*	99.6	444
JYZW12	*L. japonica*	-	1175.6	2.09	*L. japonica*	100.0	452
					L. macranthoides	100.0	452
JYZW13	*L. macranthoides*	-	49.7	1.63	*L. fulvotomentosa*	-	-
					L. hypoglauca	-	-
					L. macranthoides	100.0	452
JYZW14	*L. macranthoides*	-	740.7	2.05	*L. fulvotomentosa*	-	-
					L. hypoglauca	-	-
					L. macranthoides	100.0	452
JYZW15	*L. macranthoides*	-	1146.6	2.10	*L. fulvotomentosa*	-	-
					L. hypoglauca	-	-
					L. macranthoides	100.0	452
JYZW16	*L. macranthoides*	-	901.4	2.12	*L. fulvotomentosa*	-	-
					L. hypoglauca	-	-
					L. macranthoides	100.0	452
JYZW17	*L. macranthoides*	-	815.8	2.12	*L. fulvotomentosa*	-	-
					L. hypoglauca	-	-
					L. macranthoides	100.0	452
JYZW18	*L. macranthoides*	-	737.2	2.04	*L. fulvotomentosa*	-	-
					L. hypoglauca	-	-
JYZW19	*L. hypoglauca*	-	36.0	1.56	*L. hypoglauca*	100.0	452
JYZW20	*L. hypoglauca*	-	13.2	1.46	*L. hypoglauca*	100.0	452
JYZW21	*L. fulvotomentosa*	-	138.6	1.95	*L. fulvotomentosa*	100.0	452
YC01	Flower buds of LJF	-	412.4	2.09	*L. japonica*	100.0	452
YC02	Flower buds of LJF	-	512.4	2.10	*L. japonica*	100.0	452
YC03	Flower buds of LJF	-	277.7	2.07	*L. japonica*	100.0	452
YC04	Flower buds of LJF	-	307.0	2.07	*L. japonica*	99.6	449
YC05	Flower buds of LJF	-	187.4	2.08	*L. japonica*	100.0	442
YC06	Flower buds of LJF	-	248.0	2.07	*L. japonica*	99.6	434
YC07	Flower buds of LJF	-	342.4	2.07	*L. japonica*	100.0	452

					L. macranthoides	100.0	452
YC08	Flower buds of LJF	-	132.3	1.99	*L. fulvotomentosa*	-	-
					L. hypoglauca	-	-
					L. macranthoides	100.0	452
YC09	Flower buds of LJF	-	105.3	2.06	*L. fulvotomentosa*	-	-
					L. hypoglauca	-	-
YC10	Flower buds of LJF	-	371.1	2.14	*L. japonica*	100.0	442
YC11	Flower buds of LJF	-	282.7	2.12	*L. japonica*	99.6	444
YC12	Flower buds of LJF	-	855.0	2.18	*L. japonica*	99.6	438
YC13	Flower buds of LJF	-	626.4	2.13	*L. japonica*	100.0	442
YC14	Flower buds of LJF	-	958.3	2.13	*L. japonica*	100.0	442
YP01	Decoction pieces of LJF	-	526.6	2.06	*L. japonica*	100.0	442
YP02	Decoction pieces of LJF	-	666.9	2.05	*L. japonica*	100.0	442
YP03	Decoction pieces of LJF	-	497.9	2.04	*L. japonica*	100.0	442
YP04	Decoction pieces of LJF	-	497.2	2.09	*L. japonica*	100.0	442
YP05	Decoction pieces of LJF	-	585.6	2.05	*L. japonica*	100.0	442
YP06	Decoction pieces of LJF	-	319.1	2.02	*L. japonica*	100.0	442
YP07	Decoction pieces of LJF	-	563.6	2.03	*L. japonica*	100.0	442
YP08	Decoction pieces of LJF	-	393.1	2.08	*L. japonica*	100.0	442
YP09	Decoction pieces of LJF	-	626.8	2.05	*L. japonica*	100.0	442
					L. macranthoides	100.0	452
YP10	Decoction pieces of LJF	-	159.5	2.06	*L. fulvotomentosa*	-	-
					L. hypoglauca	-	-
YP11	Decoction pieces of LJF	-	445.7	2.07	*L. japonica*	100.0	442
					L. macranthoides	100.0	452
YP12	Decoction pieces of LJF	-	258.8	2.12	*L. fulvotomentosa*	-	-
					L. hypoglauca	-	-
YP13	Decoction pieces of LJF	-	501.1	2.09	*L. japonica*	100.0	442
YP14	Decoction pieces of LJF	-	526.2	2.12	*L. japonica*	100.0	452
YP15	Decoction pieces of LJF	-	346.6	2.15	*L. macranthoides*	100.0	452
					L. fulvotomentosa	-	-

					L. hypoglauca	-	-
YP16	Stir-fried LJF	-	530.9	2.06	*L. japonica*	100.0	452
YP17	Stir-fried LJF	-	446.0	2.10	*L. japonica*	100.0	442
YP18	Stir-fried LJF	-	449.0	2.14	*L. japonica*	100.0	452
YP19	Stir-fried LJF	-	113.2	1.97	-	-	-
YP20	Stir-fried LJF	-	332.4	2.07	*L. japonica*	100.0	442
					L. macranthoides	100.0	452
YP21	Decoction pieces of LJF	-	352.5	2.11	*L. fulvotomentosa*	-	-
					L. hypoglauca	-	-
YP22	Decoction pieces of LJF	-	114.1	2.07	*L. japonica*	99.1	430
YP23	Decoction pieces of LJF	-	524.7	2.12	*L. japonica*	100.0	452
YP24	Decoction pieces of LJF	-	503.6	2.09	*L. japonica*	99.6	444
YP25	Decoction pieces of LJF	-	520.5	2.15	*L. japonica*	99.1	436
ZCY01	Zhizi Jinhua Pills	12308121	498.9	1.71	-	-	-
ZCY02	Zhizi Jinhua Pills	12310097	1006.4	1.50	-	-	-
ZCY03	Zhizi Jinhua Pills	24081443	284.6	1.51	-	-	-
ZCY04	Zhizi Jinhua Pills	24081456	348.2	1.93	Mixture of multiple species	-	-
ZCY05	Zhizi Jinhua Pills	25022019	657.5	1.46	-	-	-
ZCY06	Yinqiao Jiedu Tablets	24124897	302.5	1.73	Mixture of multiple species	-	-
ZCY07	Yinqiao Jiedu Tablets	24124896	271.1	1.82	Mixture of multiple species	-	-
ZCY08	Yinqiao Jiedu Tablets	440143	364.3	1.97	-	-	-
ZCY09	Yinqiao Jiedu Tablets	23001	394.9	1.60	*L. japonica*	100.0	442
ZCY10	Jiannao Bushen Pills	240202	163.3	1.43	*L. japonica*	100.0	442
ZCY11	Jiannao Bushen Pills	241106	168.7	1.29	-	-	-
ZCY12	Jiannao Bushen Pills	2407001	132.8	1.77	-	-	-
ZCY13	Niuhuang Qinggong Pills	22013506	263.3	2.01	*Nelumbo nucifera*	100.0	420
ZCY14	Niuhuang Qinggong Pills	23013345	289.5	2.04	Mixture of multiple species	-	-
ZCY15	Niuhuang Qinggong Pills	240401009	78.4	1.78	-	-	-
ZCY16	Jinsang Sanjie Pills	WA124050572	213.1	1.89	*L. japonica*	100	442
ZCY17	Jinsang Sanjie Pills	WA124050562	387.7	1.90	Mixture of multiple species	-	-
ZCY18	Jinsang Sanjie Pills	WA124010062	174.6	1.91	-	-	-

ZCY19	Qingguo Pills	22035429	131.2	1.94	Mixture of multiple species	-	-
ZCY20	Qingguo Pills	240401	496.4	1.71	*Paeonia lactiflora*	100.0	450
ZCY21	Qingguo Pills	240901	711.6	1.65	-	-	-
ZCY22	Qingguo Pills	24499004	711.6	1.65	-	-	-
ZCY23	Qingguo Pills	231103	220.7	1.82	*P. lactiflora*	100.0	450

"-" indicates not applicable.

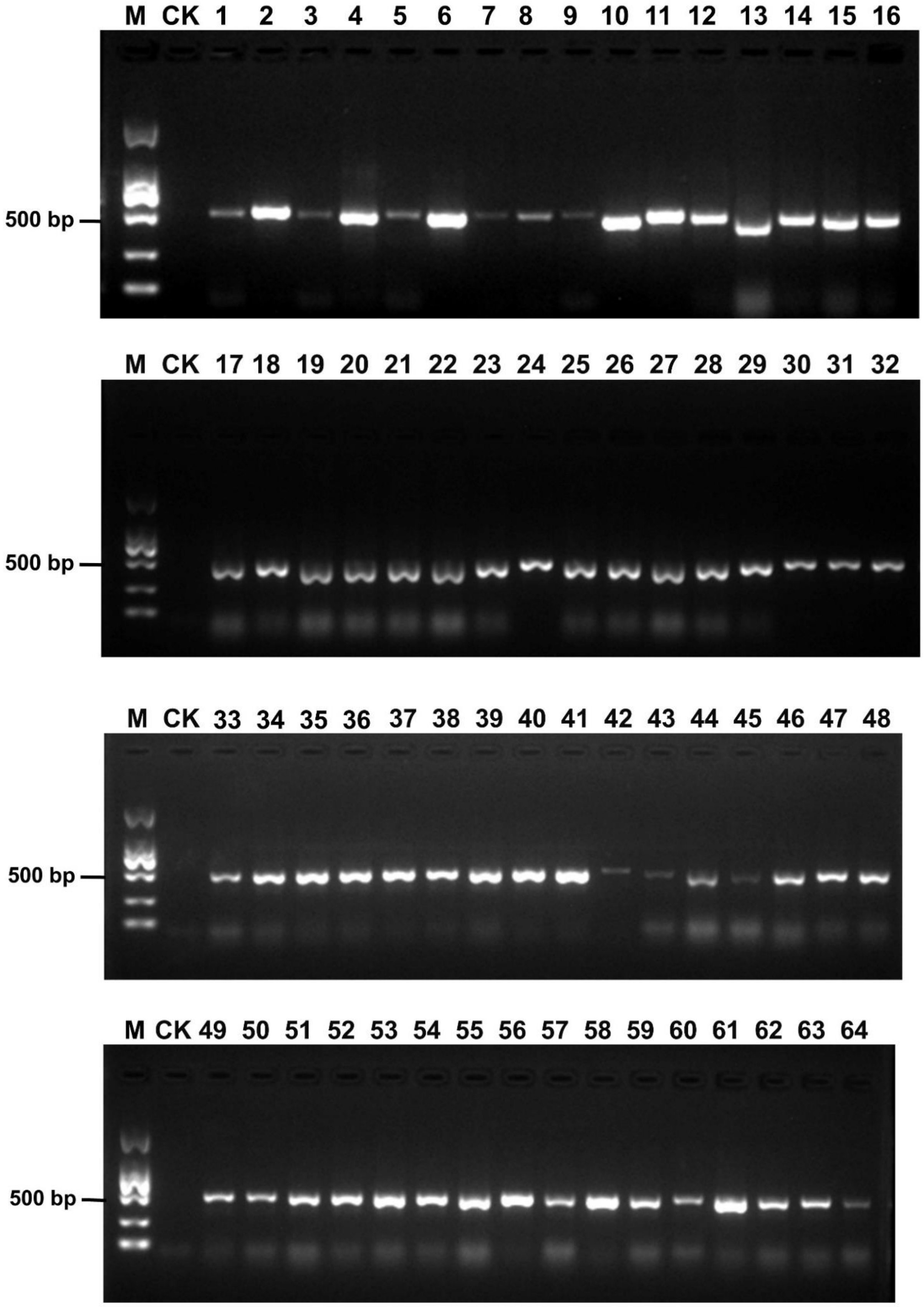
M CK 1 2 3 4 5 6 7 8 9 10 11 12 13 14 15 16
500 bp
M CK 17 18 19 20 21 22 23 24 25 26 27 28 29 30 31 32
500 bp
M CK 33 34 35 36 37 38 39 40 41 42 43 44 45 46 47 48
500 bp
M CK 49 50 51 52 53 54 55 56 57 58 59 60 61 62 63 64
500 bp

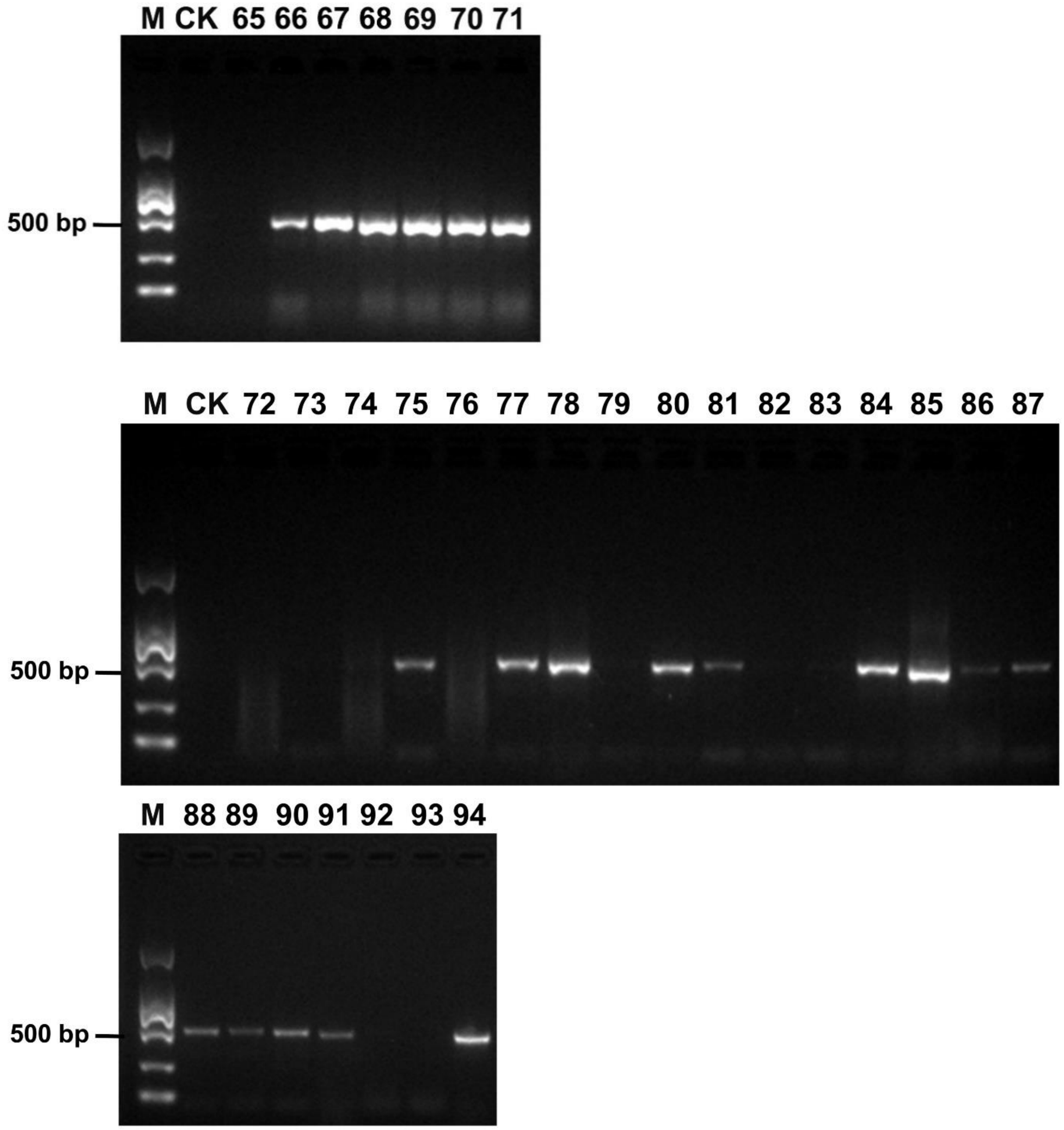

Fig. S1 Agarose gel electrophoresis showing PCR amplification products of the ITS2 sequence

M: DL 2000 DNA Marker; CK: Negative control;

1: ZZ01	2: ZZ02	3: ZZ03	4: ZZ04	5: ZZ05
6: ZZ06	7: ZZ07	8: ZZ08	9: ZZ09	10: ZZ10
11: ZZ11	12: JYZW01	13: JYZW02	14: JYZW03	15: JYZW04
16: JYZW05	17: JYZW06	18: JYZW07	19: JYZW08	20: JYZW09
21: JYZW10	22: JYZW11	23: JYZW12	24: JYZW13	25: JYZW14
26: JYZW15	27: JYZW16	28: JYZW17	29: JYZW18	30: JYZW19
31: JYZW20	32: JYZW21	33: YC01	34: YC02	35: YC03

36: YC04	37: YC05	38: YC06	39: YC07	40: YC08
41: YC09	42: YC10	43: YC11	44: YC12	45: YC13
46: YC14	47: YP01	48: YP02	49: YP03	50: YP04
51: YP05	52: YP06	53: YP07	54: YP08	55: YP09
56: YP10	57: YP11	58: YP12	59: YP13	60: YP14
61: YP15	62: YP16	63: YP17	64: YP18	65: YP19
66: YP20	67: YP21	68: YP22	69: YP23	70: YP24
71: YP25	72: ZCY01	73: ZCY02	74: ZCY03	75: ZCY04
76: ZCY05	77: ZCY06	78: ZCY07	79: ZCY08	80: ZCY09
81: ZCY10	82: ZCY11	83: ZCY12	84: ZCY13	85: ZCY14
86: ZCY15	87: ZCY16	88: ZCY17	89: ZCY18	90: ZCY19
91: ZCY20	92: ZCY21	93: ZCY22	94: ZCY23	

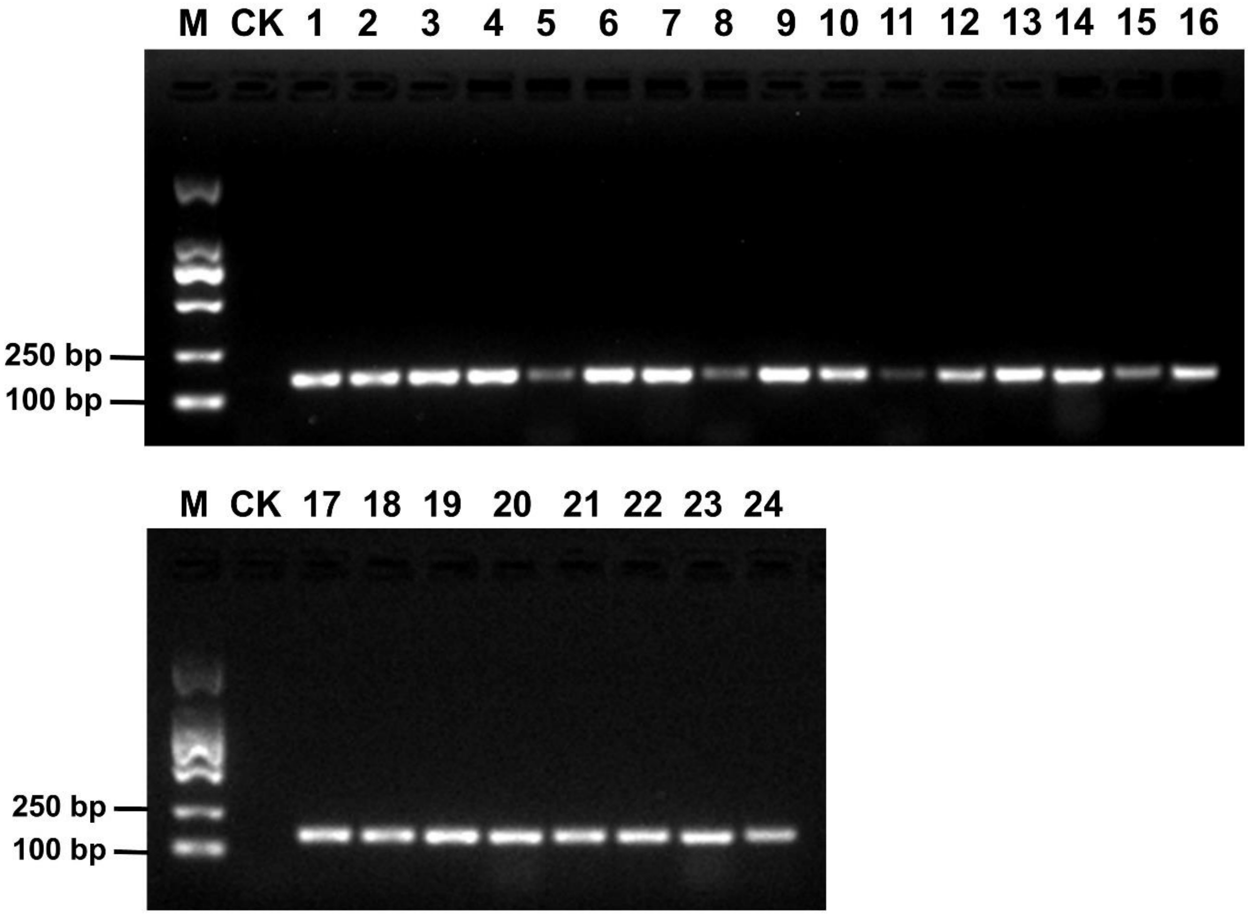

Fig. S2 Agarose gel electrophoresis showing PCR amplification products of mini-barcode

M: DL 2000 DNA Marker; CK: Negative control;

1: ZCY01	2: ZCY02	3: ZCY03	4: ZCY04	5: ZCY05

6: ZCY06	7: ZCY07	8: ZCY08	9: ZCY09	10: ZCY10
11: ZCY11	12: ZCY12	13: ZCY13	14: ZCY14	15: ZCY15
16: ZCY16	17: ZCY17	18: ZCY18	19: ZCY19	20: ZCY20
21: ZCY21	22: ZCY22	23: ZCY23	24: YP19	

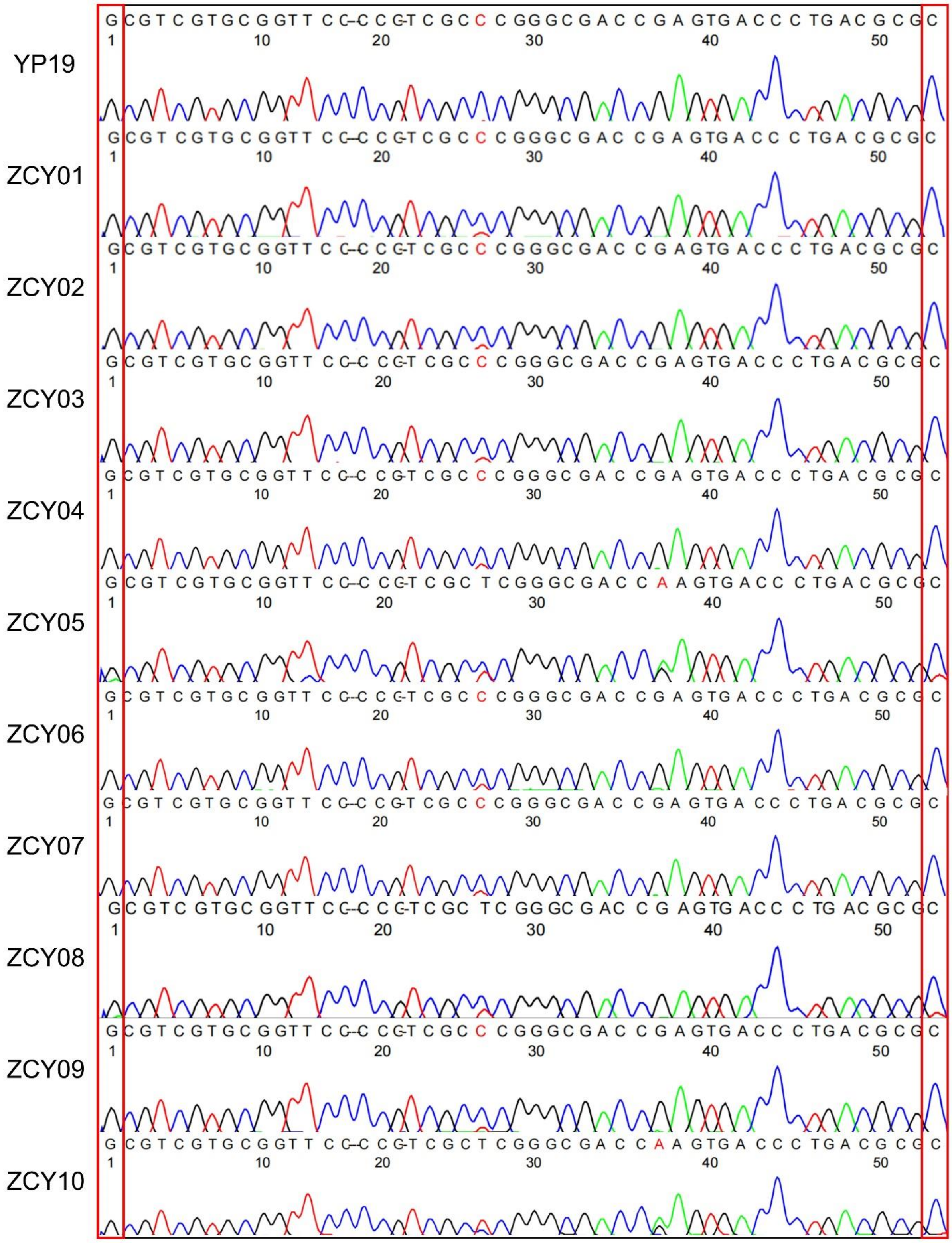
YP19
ZCY01
ZCY02
ZCY03
ZCY04
ZCY05
ZCY06
ZCY07
ZCY08
ZCY09
ZCY10

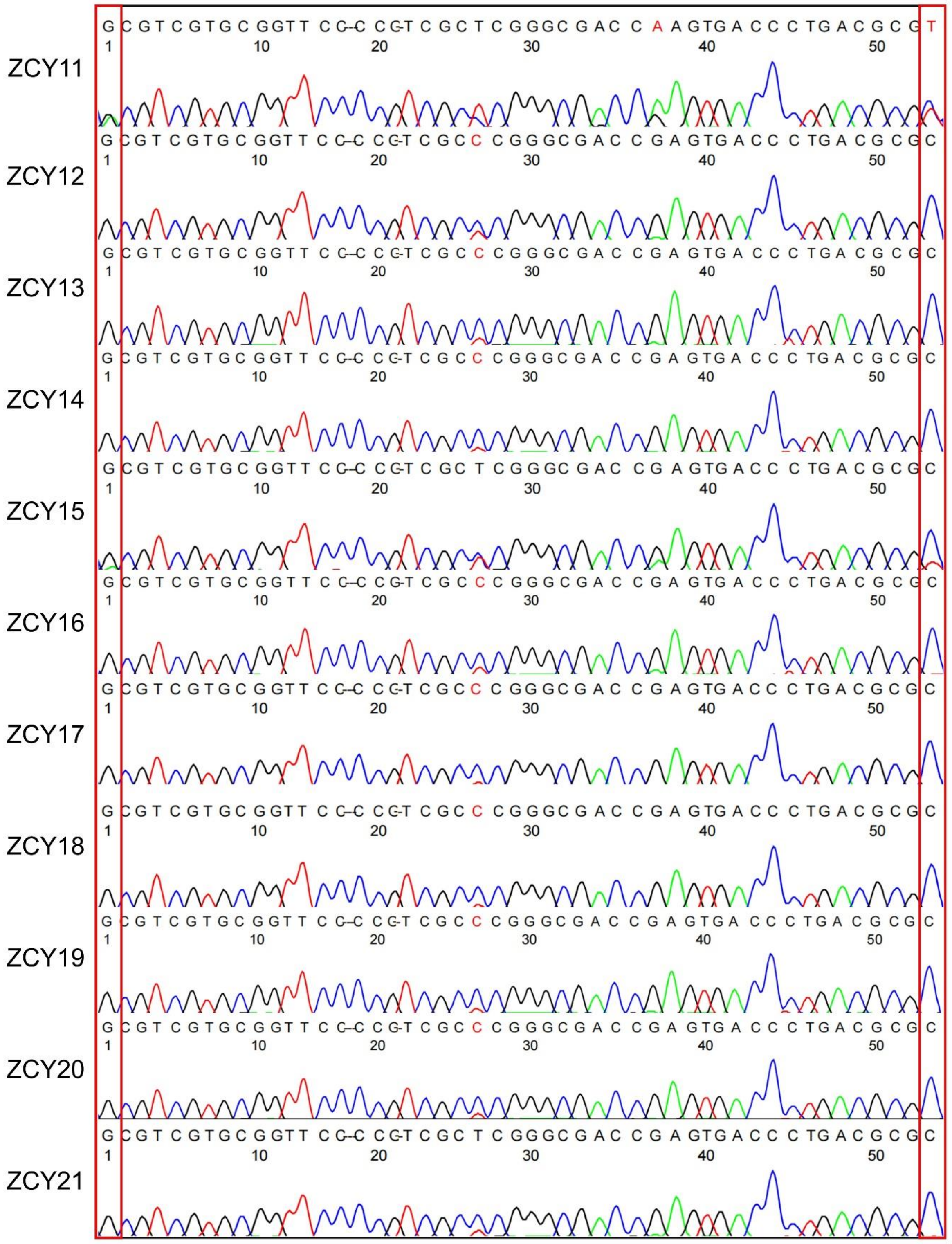
ZCY11
ZCY12
ZCY13
ZCY14
ZCY15
ZCY16
ZCY17
ZCY18
ZCY19
ZCY20
ZCY21

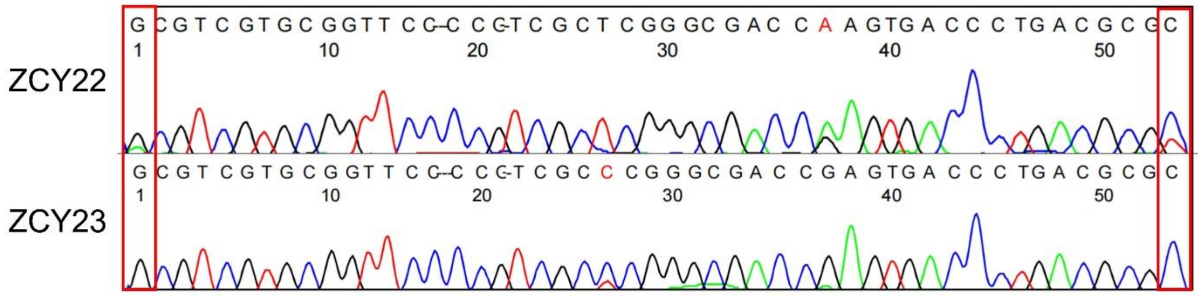

Fig. S3 Identification using mini-barcode combined with SNP double peak method. The two SNP sites are indicated within the red boxes, with the corresponding nucleotides being G/C in LJF and A/T in LF, respectively. ZCY05; ZCY08; ZCY11 ZCY15 and ZCY22 show adulteration of LF, while other samples didn't show adulteration of LF.
